# Data on calcium oxide and cow bone catalysts used for soybean biodiesel production

**DOI:** 10.1016/j.dib.2018.03.057

**Published:** 2018-03-17

**Authors:** Ayoola A. Ayodeji, Igho E. Blessing, Fayomi O. Sunday

**Affiliations:** a Chemical Engineering Department, Covenant University, Ota, Nigeria; b Mechanical Engineering Department, Covenant University, Ota, Nigeria; c Surface Engineering Research Centre, Tshwane University of Technology, South Africa

**Keywords:** Biodiesel, Calcined cow bone, Catalyst, Soybean

## Abstract

Biodiesel was produced from soybean oil using calcium oxide and cow bone as heterogeneous catalysts, through transesterification process. The soybean oil used was characterized using gas chromatography mass spectrometer (GCMS) and the cow bone catalyst produced was characterized X-ray fluorescence (XRF) spectrometer. The effects of the variation of methanol/oil mole ratio, catalyst concentration and reaction temperature on biodiesel yield during the transesterification of soybean oil were investigated. Reaction time of 3 h and stirring rate of 500 rpm were kept constant. Using Response Optimizer (Minitab 17), the optimum conditions for biodiesel production were established. It was observed that the calcination of cow bone catalyst enhanced its conversion to apatite-CaOH. Also, the results obtained showed that the performance trends of calcined cow bone catalyst and the conventional CaO catalyst were similar.

**Table:** 

Subject area	*Materials Science Engineering*
More specific subject area	*Renewable Energy*
Type of data	*Table, image*
How data was acquired	The physio-chemical characteristics (chemical compositions) of the uncalcined cow bone catalyst, calcined cow bone catalyst and CaO catalyst were determined using XRF spectroscopy principle. The fatty acids profile in the soybean oil used was analysed using GCMS. Experimental work involving transesterification process (using Box Benkhen design) was employed in generating data on biodiesel yield. Laboratory tests to generate properties of both the soybean oil used and soybean biodiesel produced were carried out.
Data format	Raw, Analyzed
Experimental factors	The cow bone sample was calcined and a portion was left uncalcined. Parameters varied during biodiesel production (transesterification process) are methanol/oil mole ratio, catalyst concentration and reaction temperature.
Experimental features	Oil sample was introduced into GCMS at oven temperature of 60 °C using 99.99% Helium with column length of 30 m, column thickness of 0.25 µm and internal diameter of 0.25 mm. The column temperature was programmed to increase to 200 °C at the rate of 10 °C per minute. The flame ionization detector (FID) temperature was set at 220 °C.
	Determination of the elemental composition of unclacined and calcined cow bone catalysts involved the use of XRF analysis. Phillips 1404 XRF Wavelength Disperse Spectrometer coupled with X-ray tube and a Rh anode (X-rays source) having HVPS 60 kV, 7.0 mA, a LN_2_ cooled Si(Li) detector with a resolution of 131 eV at, Mn Kα (5.9 keV) X-ray and a 6-sample turret (that permits the mounting and analyzing of 6 samples at a time) was used. XRF spectrometer operation was based on the emission of the excited elemental components of the given sample through the bombarding of the sample with high energy X-rays.
	During biodiesel production, methanol/oil mole ratio of 9–15, catalyst concentration of 10–20 wt/wt% and reaction temperature of 55–65 °C were considered. Reaction time of 3 h and a stirring rate of 500 rpm were kept constant.
Data source location	Department of Chemical Engineering, Covenant University, Ota, Nigeria and Metallurgical and Chemical Engineering Department, Amadu Bello University, Zaria, Kaduna State, Nigeria.
Data accessibility	Data are available within this article.

**Value of the data**

• The data on biodiesel production can be modelled to examine the relationship between the process variable (for instance methanol/oil mole ratio, catalyst concentration and reaction temperature) as it affects the yield of biodiesel.
• The data could be used at investigating the relationship between the fatty acid profiles of soybean oil and the yield of biodiesel.
• The given data will show authors in the field of material science and chemical engineering that the calcination of cow bone will enhance cow bone conversion to apatite-CaOH catalyst (Ca_10_P_6_O_26_H_2_) for biodiesel production.
• The data obtained for both the calcined and uncalcined cow bone catalyst can be used as inference to determine the chemical composition of other animal bones, under the same experimental conditions.
• The data reveals that calcined cow bone catalyst is a very promising heterogenous catalyst that can be used in the place of the conventional CaO catalyst during oil transesterification.

## 1 Data

**Fig. 1:**
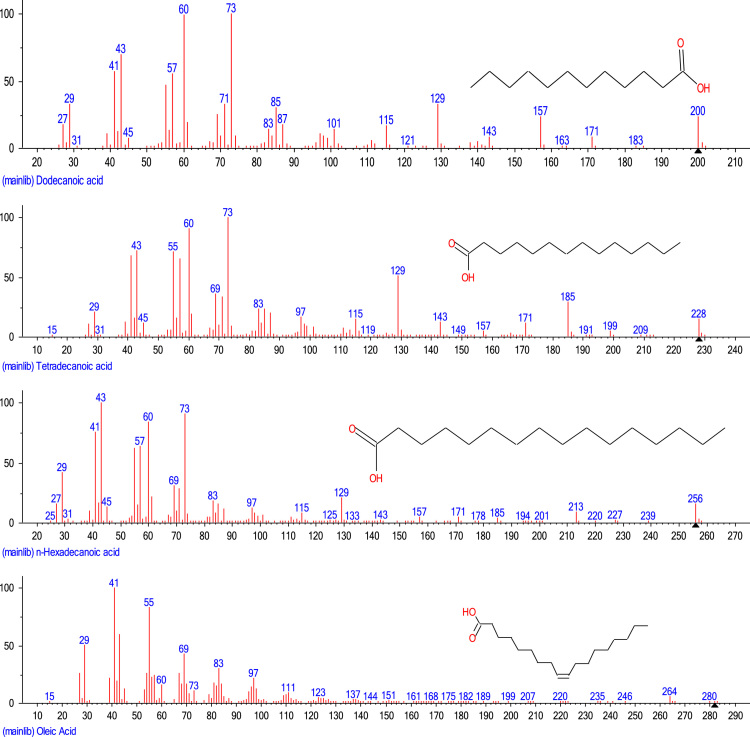
Fatty acids profile of soybean oil.

Fatty acids profile of soybean oil useful for the determination of methanol/oil mole ratio (a process parameter) was determined (Fig. 1). Table 1 shows the data obtained from XRF spectrometry of CaO, uncalcined cow bone catalyst and calcined cow bone catalyst. Data obtained from the design of experiment and the biodiesel yield obtained (using the conventional CaO and calcined cow bone catalysts) is shown in Table 2. Properties of soybean oil and soybean biodiesel are tabulated in Table 3.

**Table 1:** XRF spectrometry of CaO, Uncalcined cow bone and calcined cow bone catalysts.

Compound	Catalyst Chemical Composition (%)		
	CaO	Uncalcined cow bone	Calcined cow bone
P_2_O_5_	–	39.200	34.200
CaO	99.230	60.060	64.890
MnO-	0.013	0.010	
TiO_2_	0.010	0.020	0.055
Fe_2_O_3_	0.110	0.232	0.255
CuO	0.029	0.005	0.006
ZnO	–	–	0.022
SrO	0.120	0.130	0.143
ZrO_2_	0.010	0.008	0.007
BaO	0.240	0.180	0.160
La_2_O_3_	0.130	–	–
CeO_2_	0.040	–	–
Pr_2_O_3_	0.040	–	–
Yb_2_O_3_	0.050	–	–
Sm_2_O_3_	–	0.010	0.036
Tm_2_O_3_	–	0.120	0.140
Eu_2_O_3_	–	–	0.020
Re_2_O_7_	–	–	0.030

**Table 2:** Experimental design and biodiesel yields obtained from the transesterification process.

Catalyst Conc.	Methanol/Oil	Rxn Temp.	Biodiesel Yield	Biodiesel Yield
(wt/wt %)	mole ratio	(°C)	using CaO (%)	using calcined bone (%)
15	12	60	78.6	76.2
10	12	65	84.6	82.4
10	15	60	79.2	70.4
20	12	55	76.8	72.8
15	9	55	93.0	92.2
10	9	60	92.4	86.6
15	9	65	94.8	89.6
15	12	60	78.4	86.8
10	12	55	78.6	72.6
20	15	60	82.8	76.4
20	12	65	69.0	65.4
15	15	55	90.6	86.2
20	9	60	86.4	85.0
15	15	65	82.4	78.5
15	12	60	75.6	71.6

**Table 3:** Properties of soybean oil used and soybean biodiesel produced.

Sample	Density @ 25 °C (g/cm^3^)	Pour Point (°C)	Flash point (°C)	Viscosity @ 40 °C (mm²/s)	Water content (%)	Acid Value (mg KOH/g)
Soybean oil	0.9120	−9	237	31.73	0.072	1.072
Soybean biodiesel	0.8815	−6	205	4.65	0.006	0.064

## 2 Experimental design, materials and methods

Response surface experimental design (Box-Behnken method, Minitab 17 software) was employed to investigate the effects of variation of methanol-oil mole ratio, catalyst concentration and reaction temperature on biodiesel yield. Materials used include methanol (99% purity, Romil Ltd UK), CaO (99.5%, Qualikems, India), waste cow bones and soybean oil. Equipment used include gas chromatography mass spectrometer (Agilent Technologies 7890A GC System USA) for the determination of fatty acids profile of soybean oil, X-ray fluorescence (XRF) spectrometer for the identification and quantification of the elemental composition of CaO, uncalcined and calcined cow bones used.

The waste cow bones were first washed in clean water to remove any dirt present. They were then crushed (using electrical bone crushing machine) and grinded into small particles which were then cooked for 4 h at 100 °C using a pressure pot containing water. The water used was replaced every 30 min to remove tissues, fat and any other impurity that might be present. The hot water was then completely removed and the bone chips were then dried in an oven at 105 °C for 1 h. The dry chips obtained were then further grinded into fine powder from using electric grinder to have a particle size of 100 µm. The fine powder obtained was calcined at temperature of 800 °C in an electric furnace for 4 h to ensure complete transformation of calcium compound into apatite [1], [2], [3].

Determination of the elemental composition of unclacined and calcined cow bone catalysts involved the use of XRF analysis. Phillips 1404 XRF Wavelength Disperse Spectrometer coupled with X-ray tube and a Rh anode (X-rays source) having HVPS 60 kV, 7.0 mA, a LN_2_ cooled Si(Li) detector with a resolution of 131 eV at, Mn Kα (5.9 keV) X-ray and a 6-sample turret (that permits the mounting and analyzing of 6 samples at a time) was used. XRF spectrometer operation was based on the emission of the excited elemental components of the given sample through the bombarding of the sample with high energy X-rays.

Oil sample was introduced into GCMS at oven temperature of 60 °C using 99.99% Helium with column length of 30 m, column thickness of 0.25 µm and internal diameter of 0.25 mm. The column temperature was programmed to increase to 200 °C at the rate of 10 °C per minute. The flame ionization detector (FID) temperature was set at 220 °C. The fatty acid composition of the soybean oil used is shown in Fig. 1. The result obtained showed the presence of lauric acid (C12:0, 7%), myristic acid (C14:0, 10%), palmitic acid (C16:0, 14%), oleic acid (C18:1, 56%) and others (13%).

The chemical compositions of CaO, uncalcined cow bone catalyst and calcined cow bone catalyst are shown in Table 1. Biodiesel was produced using transesterification process as reported in [4]. The transesterification process for the biodiesel yield obtained is shown in Table 2. Optimum conditions obtained during the transesterification process (for both CaO and calcined cow bone catalysts) are reaction temperature of 60 °C, methanol/oil mole ratio of 10 and catalyst concentration of 18 wt/wt Oil. Comparatively, difference in biodiesel yield was small using CaO and calcined cow bone catalyst. The advantages of the use of the readily available calcined cow bone (as catalyst) include its availability, low cost of production and similar performance trend (compare to the conventional CaO catalyst), during the transesterification of soybean oil. Table 3 provides the properties of soybean oil used and soybean biodiesel produced.
